# Neuroprotective effects of the combined treatment of resveratrol and urapidil in experimental cerebral ischemia-reperfusion injury in rats

**DOI:** 10.1590/acb395329

**Published:** 2024-08-05

**Authors:** Rıdvan Çetin, Sinan Bahadir, İbrahim Basar, Barış Aslanoglu, Burak Atlas, Seval Kaya, Barış Can Güzel, Yahya Turan

**Affiliations:** 1 Dicle University – Faculty of Medicine – Department of Neurosurgery – Diyarbakır – Turkey.; 2 Baskent University – Faculty of Medicine – Department of Neurosurgery – Ankara – Turkey.; 3 Istanbul Aydin University – Faculty of Medicine – Department of Histology and Embryology – Istanbul – Turkey.; 4 Siirt University – Department of Veterinary Anatomy – Siirt – Turkey.

**Keywords:** Brain Ischemia, Resveratrol, Urapidil, Oxidative Stress, Apoptosis, Rats

## Abstract

**Purpose:**

To evaluate the neuroprotective effect of resveratrol, urapidil, and a combined administration of these drugs against middle cerebral artery occlusion (MCAO) induced ischemia/reperfusion (IR) injury model in rats.

**Methods:**

Thirty-five rats were divided into five groups of seven animals each. Animals in IR, IR resveratrol (IRr), IR urapidil (IRu), and IR + combination of resveratrol and urapidil (IRc) were exposed to MCAO induced cerebral ischemia reperfusion injury model. Rats in IRr and IRu groups received 30-mg/kg resveratrol and 5-mg/kg urapidil respectively. Animals in IRc received a combined treatment of both drugs. At the end of the study, brain tissues were used for oxidative stress (malondialdehyde, glutathione, and superoxide dismutase), pro-apoptotic caspase-3, anti-apoptotic Bcl-2, and pro-inflammatory tumor necrosis factor-α cytokine level measurements.

**Results:**

The MCAO model successfully replicated IR injury with significant histopathological changes, elevated tissue oxidative stress, and upregulated apoptotic and inflammatory protein expression in IR group compared to control group (*p* < 0.001). All parameters were significantly alleviated in IRr group compared to IR group (all *p* < 0.05). In IRu group, all parameters except for caspase-3 and Bcl-2 were also significantly different than IR group (all *p* < 0.05). The IRc group showed the biggest difference compared to IR group in all parameters (all p < 0.001). The IRc had higher superoxide dismutase and Bcl-2 levels, and lower caspase-3 levels compared to both IRr and IRu groups (all p < 0.05). Also, the IRc group had lower MDA and TNF-α levels compared to IRu group (all p < 0.05).

**Conclusions:**

The results indicate that combined treatment of resveratrol and urapidil may be a novel strategy to downregulate neurodegeneration in cerebral IR injury.

## Introduction

Stroke, caused by cerebral ischemia, is the second reason for mortality around the world, and it is responsible for 10.8 to 12.2% of all deaths in low- and middle-income countries^1^.

Reperfusion, providing blood to the ischemic brain, is the sole accepted treatment despite the risk of excessive reactive oxygen species (ROS) and/or reactive nitrogen species (RNS) production, leading to ischemia/reperfusion (IR) injury^2^. The accumulation of excessive ROS and RNS is a key factor in tissue dysfunction in IR injury, leading to the induction of apoptotic cell death and inflammation in related organs^3^^,^ 4.

Although numerous experimental and clinical studies reported promising neuroprotective effects of various substances, the dose-dependent adverse effects of neuroprotective drugs might be a limitation reason for the approval of these modalities^5^.

Resveratrol is one of the most widely studied antioxidants in cerebral IR injury^6^. It also gained attention of the researchers in other diseases such as cancer, hypertension, and diabetes due to its safety, easy accessibility, and multifunctional properties^7^. Experimental studies have shown that resveratrol treatment alleviates IR injury in different organs^8^^,^ 9. Moreover, previously published studies reported promising results of resveratrol administration to alleviate neurodegeneration in cerebral IR injury^10^.

Urapidil, an anti-hypertensive agent, has demonstrated beneficial effects against IR injury in various organs, regulating oxidative stress, apoptotic signaling, and inflammation in experimental studies^11^^,^ 12. However, to the best of our knowledge, the neuroprotective effect of urapidil in cerebral IR injury has not been studied previously.

This study aimed to examine the neuroprotective effects of resveratrol and urapidil, both individually and in combination, in cerebral IR injury in rats.

## Methods

### Study design

All experimental protocols of this study were performed after approval of local Experimental Animal Ethics Committee of Dicle University (Approval no. 2021/40), in February 24, 2022.

Thirty-five female mature Sprague Dawley rats were randomly divided into five groups–control, IR, IR resveratrol (IRr), IR urapidil (IRu), and IR + combination of resveratrol and urapidil (IRc)–of seven animals in each. Animals in control group were not exposed to any surgical procedure. Rats in IR, IRr, IRu, and IRc groups were exposed to middle cerebral artery occlusion to induce stroke. For this, the animals were anesthetized by ketamine hydrochloride (80 mg/kg i.m.) (Ketalar; Pfizer, Istanbul, Turkey) and xylazine hydrochloride (10 mg/kg i.m.) (Rompun; Bayer Healthcare, Leverkusen, Germany).

Following anesthesia, each animal was administered cefazolin (50 mg/kg i.m.) prior to surgical procedure. The middle cerebral artery occlusion (MCAO) model was performed as described previously^13^. In short, following general anesthesia, right common, carotid artery (CCA), external carotid artery (ECA), and internal carotid artery (ICA) were exposed by thorough dissection in the neck. Following clamping of CCA, a 3-0 monofilament suture was inserted through a small incision towards ICA bifurcation. After occluding right middle cerebral artery by this method for 2 h, the suture was removed to allow reperfusion. Then, incisions were sutured, and rats were allowed to recover.

Animals in treatment groups (IRr, IRu, and IRc) were administered intraperitoneal treatments at the 0th, 24th, 48th, and 72nd h of reperfusion. Animals in IRr received 30-mg/kg resveratrol, and the animals in IRu received 5-mg/kg urapidil. Animals in IRc group received both resveratrol (30 mg/kg) and urapidil (5 mg/kg) in combination. The rats received no additional analgesic or antimicrobial agent postoperatively.

All the animals were sacrificed at the 72nd h of experiment after receiving final doses. Brain tissue samples were extracted and used for both biochemical and histopathological analyses. Biochemical samples were stored at -80ºC, and histopathological samples were fixed in 10% buffered formaldehyde for routine tissue processing protocol.

### Measurement of tissue malondialdehyde, glutathione, and superoxide dismutase

Tissue malondialdehyde (MDA) levels were measured for the detection of lipid peroxidation^14^^,^ 15. For that purpose, a method previously described by Placer et al.^14^ was used, and the results are expressed as nmol/g MDA tissue.

Tissue reduced glutathione (GSH) levels were examined as previously described methods, and the results are expressed as nmol/g GSH tissue^16^.

Tissue levels of superoxide dismutase (SOD) were analyzed as Misra et al. described, and the results are expressed as U/mg protein^17^.

### Tissue preparation process

Tissue samples were processed as described previously^18^. The previously fixed tissue samples were washed under tap water and dehydrated through increasing alcohol series. Then, they were immersed in xylene for clearing and embedded into paraffin block. Five-µm thick samples were obtained with a rotary microtome, and these samples were stained with hematoxylin and eosin for pathological examinations.

### Immunohistochemistry protocol

Apoptosis related caspase-3 and Bcl-2, as well as pro-inflammatory cytokine tumor necrosis factor (TNF)-α levels, were measured with immunohistochemistry. The sections to be used for immunochemistry were deparaffinized in xylene, re-hydrated through decreasing alcohol series and washed in phosphate buffered saline (PBS). Antigen retrieval was performed in citrate buffer (pH = 6), endogenous peroxidase activity is inhibited in 3% H_2_O_2_ solution, which is prepared in methanol, and non-specific binding was blocked with a ready to use blocking solution. The antibodies of caspase-3 (Cat. No.: sc-56053, Santa Cruz Biotechnology, Dallas, TX, United States of America), Bcl-2 (Cat. No.: sc-7382, Santa Cruz Biotechnology, Dallas, TX, United States of America), and TNF-α (Cat. No.: sc-52746, Santa Cruz Biotechnology, Dallas, TX, United States of America) were diluted in dilution buffer with 1:150, 1:100, and 1:200 ratios, respectively.

Prepared antibody samples were dropped onto the sections and incubated at +4ºC for overnight. Secondary antibody and enzyme binding protocols were performed with a commercially produced ready-to-use kit (Cat No.: TP-125-HL; Thermo Scientific, Waltham, MA, United States of America), and all steps were performed as described previously^19^. All sections were counterstained with hematoxylin and mounted with Entellan (Merck, Germany). Prepared samples were visualized under a light microscope.

### Quantification of immunodensity

Immunodensity of the interested proteins was analyzed with ImageJ software (National Institutes of Health, MA, United States of America). For that purpose, randomly selected three regions from each animal’s brain section (in total, 21 areas per group) were analyzed as described previously^20^^,^ 21. 3,3’-Diaminobenzidine (DAB) immunopositivity in cortical structure was compared with tissue section area, and the obtained immunodensity measurements were analyzed statistically.

### Statistical analysis

The biochemical and immunodensity results were analyzed statistically to determine whether there was any statistical difference between the groups. Statistical Package for the Social Sciences version 24.0 and GraphPad Prism 9.0.2 software were used for statistical analysis and demonstration of the results. Normality analysis was performed by the Shapiro-Wilk’s test. For statistical analysis, one-way analysis of variance (ANOVA) and Kruskal-Wallis’ tests were used, depending on the distribution of data. Comparisons between group pairs were performed with post-hoc Tukey’s test and Dunn’s test with Bonferroni correction, respectively. Results are expressed as mean ± standard deviation, and *p* < 0.05 was considered as significant.

## Results

### Biochemical results

Findings regarding tissue MDA, GSH, and SOD levels are shown in Table 1. ANOVA test revealed a statistical difference between groups (*p* < 0.001).

**Table 1:** Biochemical measurements of MDA, GSH, and SOD levels in brain tissues.

Study Group	MDA (nmol/g tissue)	GSH (nmol/g tissue)	SOD (U/mg protein)
Control	15.80 ± 2.50	1.59 ± 0.36	106.77 ± 8.86
IR	22.83 ± 3.01*	0.71 ± 0.29*	56.30 ± 20.50*
IRr	17.32 ± 1.89	1.35 ± 0.34	86.23 ± 10.78
IRu	18.57 ± 2.53	1.32 ± 0.42	81.10 ± 16.28
IRc	14.27 ± 2.05**	1.68 ± 0.47	108.40 ± 7.06***

MDA: malondialdehyde; GSH: glutathione; SOD: superoxide dismutase; IR: ischemia/reperfusion;

* *p* < 0.05 compared to all other groups;

** *p* < 0.05 compared to IRu;

*** *p* < 0.05 compared to IRr and IRu.

Source: Elaborated by the authors.

Post-hoc analysis indicated that tissue MDA levels were significantly higher (*p* < 0.001) in the IR group, compared to other groups (all *p* < 0.05). The MDA levels in treatment groups (IRr, IRu, and IRc) were similar to the control group (all *p* > 0.05). IRr and IRu groups didn’t differ from each other (*p* > 0.05). The IRr group was also similar to IRc group (*p* > 0.05), while the IRu was significantly higher (*p* < 0.05).

The results of GSH levels analysis were similar to the ones of MDA levels. The lowest GSH level was observed in IR group, which was significantly different from the rest (all *p* < 0.05). All the treatment groups were similar to the control group (all *p* > 0.05). Similarly, none of the treatment groups showed difference from each other (all *p* > 0.05).

SOD levels in IR group were significantly lower than the control and treatment groups (all *p* < 0.05). Though IRr and IRc groups were similar to control group (both *p* < 0.05), the IRu group was significantly lower compared to control group (*p* > 0.05). SOD levels in IRc group was significantly higher than both IRr and IRu groups (both *p* < 0.05).

**Figure 1:**
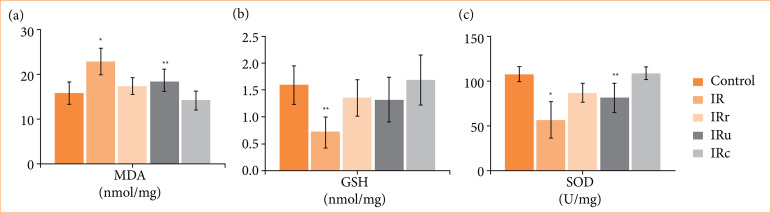
Statistical results of **(a)** MDA analysis: *p < 0.005 in IR group compared to control, IRr, and IRc groups, **p < 0.05 in IRu group compared to IR and IRc groups; **(b)** GSH analysis: **p < 0.05 in IR group compared to other groups; **(c)** SOD analysis: *p < 0.005 in IR group, compared to control, IRr, and IRc groups, **p < 0.05 in IRu group compared to control, IR and IRc groups.

MDA: malondialdehyde; GSH: glutathione; SOD: superoxide dismutase; IR: ischemia/reperfusion. Source: Elaborated by the authors.

Graphical demonstration of the statistical analysis is shown in Fig. 1. Statistical analyses of histopathological parameters are summarized in Table 1. The detailed results of post-hoc Tukey’s tests are given in Suppl. Tables 1–3^22^.

### Histopathological findings

Microscopic examinations demonstrated severe injury in cerebral substantia grisea and alba following cerebral IR. Widespread neuron and glia cell nuclear pyknosis were present. In the IR group, tissue loss in the cerebral cortex was apparent. Excessive edema was observed in the substantia alba. Furthermore, in the white matter, there was a significant disorganization of nerve fibers and widespread irregularities in neural cell bodies due to edema.

In the IRr group, besides the pyknotic nuclei in neurons and glia cells, most of the neurons and glial cells in this group were normal. There was edema in the parenchyma.

In the IRu group, the mentioned pathological degenerations were alleviated significantly. In cerebral cortex, pyknotic neuron and glia cell nuclei were observed seldomly. Perineural and perivascular edema in this group was not prominent as in IR group.

The morphological degenerations were significantly alleviated in IRc group. The tissues showed similar morphology to the one observed in control group.

**Figure 2:**
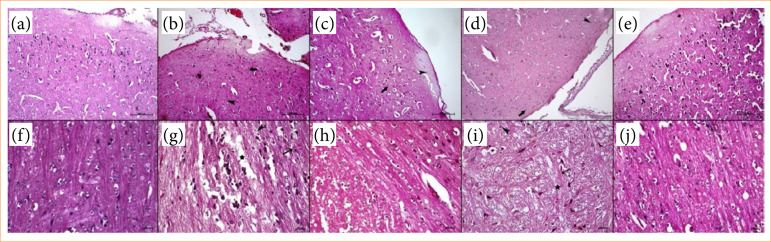
Representative histopathological sections of **(a–e)** the cerebral cortex and **(f–j)** white mater in **(a–f)** control, **(b–g)** ischemia/reperfusion (IR), **(c–h)** IRr, **(d–i)** IRu, and **(e–j)** IRc groups. Nuclear pyknosis in neurons (arrowhead), and glial cells (thick arrow). Desquamated and edema accumulated neuronal parenchyma in white mater (star). Staining: hematoxylin and eosin, Bar: **(a–e)** 200 µm, **(f–j)** 20 µm.

Source: Elaborated by the authors.

Representative histopathological micrographs of the groups are shown in Fig. 2.

### Immunohistochemistry results

**Figure 3:**
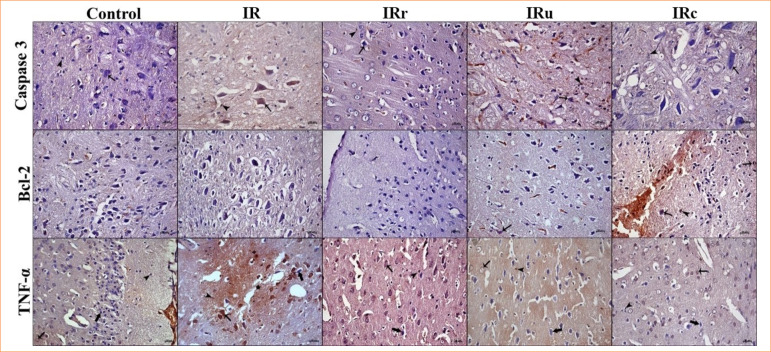
Representative immunodensity micrographs of caspase-3, Bcl-2 and TNF-α in control, IR, IRr, IRu, and IRc groups. Immunoexpression of caspase-3 was low in neurons (arrow) in control group. The caspase-3 expression is dramatically upregulated in neurons (arrow) and glial cells (arrowhead) of IR group. Tissue caspase-3 immunopositivity in neural cells (arrow) and neuroglia cells (arrowhead) in IRr and IRu groups were significantly downregulated, and the neural cell (arrow) and neuroglia (arrowhead) immunopositivty of caspase-3in IRc group was similar to control group. The Bcl-2 immunopositivity is significantly suppressed in IR group. The neuron cell immunopositivity in treatment groups (arrow) were significantly upregulated, and the most immunodensity was observed in IRc group. The TNF-α immunopositivity in neuroglia cells (arrow) and brain tissue (arrow). Counterstaining: hematoxylin, bar: 20 µm.

Representative immunohistochemistry micrographs of the groups are shown in Fig. 3. ANOVA test revealed a statistical difference between groups regarding Bcl-2 (*p* < 0.001), and Kruskal-Wallis’ test showed statistical difference in terms of caspase-3 and TNF-α (both *p* < 0.001).

TNF-α: tumor necrosis factor-α; IR: ischemia/reperfusion. Source: Elaborated by the authors.

The experimental model resulted in significant upregulation in caspase-3 and TNF-α expression and downregulation of Bcl-2 expression. The IR group showed identical pattern in terms of all caspase-3 and Bcl-2, being significantly different than control, IRr, and IRc groups (all *p* < 0.05), and similar to IRu group (*p* > 0.05). In terms of TNF-α, IR group was different from all other groups (all *p* < 0.05).

The highest caspase-3 immunodensity was observed in IR group, and results of this group were significantly different (*p* < 0.05) than in control, IRr, and IRc groups, but not IRu. Additionally, the caspase-3 immunodensity in IRc group was similar (*p* > 0.05) to the control group, while significantly lower (*p* < 0.05) than IR and other treatment groups (*p* < 0.05).

Regarding Bcl-2, IRr and IRu groups were not different from each other and Control group (all *p* > 0.05). On the other hand, IRc group had significantly higher Bcl-2 density compared to other treatment groups, as well as the control group (all *p* < 0.05).

Similar to seen in caspase-3, IRu group had significantly higher TNF-α density than both the control and the IRc groups (both *p* < 0.05). Control, IRr, and IRc groups did not differ from each other (all *p* < 0.05).

The tissue TNF-α level was highest in IR group, and results of immunodensity in this group were significantly different (*p* < 0.05) than all other groups. Control, IRr and IRc groups were similar to each other (*p* > 0.05). IRu group had a similar Bcl-2 density to IRr (*p* > 0.05), but higher than IRc group (*p* < 0.05).

**Figure 4:**
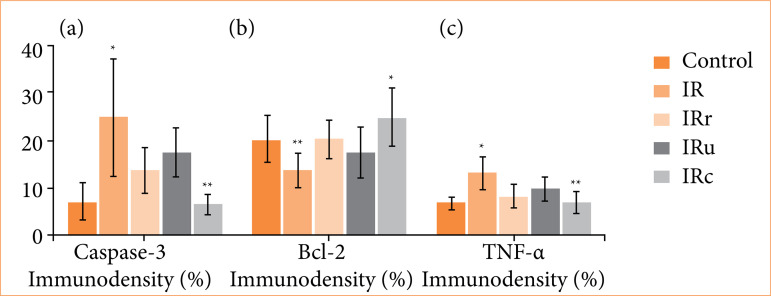
Statistical analysis results of **(a)** caspase-3 immunodensity analysis: *p < 0.05 in IR group compared to control, IRr, and IRc groups; **p < 0.001 in IRc group compared to IRr and IRu groups. **(b)** Bcl-2 immunodensity analysis: **p < 0.001 in IR group compared to control, IRr, and IRu groups, *p < 0.05 in IRc group compared to IRr and IRu groups. **(c)** TNF-α immunodensity analysis: *p < 0.05 in IR group compared to control, IRr, IRu, and IRc groups, **p < 0.05 in IRc group compared to IRu group.

A graphical demonstration of the statistical analysis is shown in Fig. 4. Statistical analysis of histopathological parameters is summarized in Table 2. The detailed results of post-hoc Tukey’s tests are given in Suppl. Tables 4–6^22^.

TNF-α: tumor necrosis factor-α; IR: ischemia/reperfusion.Source: Elaborated by the authors.

**Table 2:** Immunodensity measurements of caspase-3, Bcl-2, and TNF-α.

Study Group	Caspase-3 Immunodensity (%)	Bcl-2 Immunodensity (%)	TNF-α Immunodensity (%)
Control	7.16 ± 3.99	20.33 ± 5.12	6.79 ± 1.44
IR	25.00 ± 12.47*	13.8 ± 3.63*	13.18 ± 3.53**
IRr	13.74 ± 5.00	20.4 ± 4.05	8.34 ± 2.50
IRu	17.59 ± 5.15***	17.56 ± 5.42	9.8 ± 2.60***
IRc	6.56 ± 2.22****	25.00 ± 6.26**	7.08 ± 2.37

TNF-α: tumor necrosis factor-α; IR: ischemia/reperfusion;

* *p* < 0.05 compared to control, IRr, and IRc;

** *p* < 0.05 compared to all other groups;

*** *p* < 0.05 compared to control and IRc;

**** *p* < 0.05 compared to IR, IRr, and IRu.

Source: Elaborated by the authors.

## Discussion

This study demonstrated the effectiveness of resveratrol and urapidil against oxidative damage following IR injury, albeit at different levels. Furthermore, the combined use of resveratrol and urapidil exhibited superior efficacy compared to urapidil by itself, as evidenced by MDA and SOD levels. Additionally, it surpassed the effectiveness of resveratrol alone, particularly in SOD levels. Similarly, the combined treatment exhibited superior antiapoptotic and anti-inflammatory effects when compared to the individual administration of resveratrol or urapidil.

Cerebral stroke is recognized as one of the most common reasons of disabilities and death^23^. Around 85% of cerebral strokes are associated with cerebral ischemia, caused by an embolism or thrombus in a major cerebral artery leading to decreased or ceased blood flow to the cerebrum^24^. In clinical practice, there is no widely effective treatment for cerebral stroke except for thrombolytic recombinant tissue plasminogen activator. This therapeutic agent must be administered within a critical time frame of 3 to 4.5 h after the onset of a stroke^25^. However, with the development of experimental cerebral injury models, researchers examined numerous strategies to improve the recovery of brain tissue in cerebral stroke. These studies have primarily explored antioxidant or vasodilatory substances to mitigate the neurodegenerative effects of hypoxia in cerebral structures^26^^,^ 27.

Resveratrol, an antioxidant agent, is known to exert its protective effects against cerebral ischemia not only by its antioxidative properties, but also by its vasodilatory and anti-atherosclerotic properties^28^. It has been also shown that neuroprotective effects of resveratrol may increase when used in conjunction with other agents. For instance, Liu et al. demonstrated that combined pre-treatment with rosuvastatin and resveratrol was more effective against IR injury in rats compared to rosuvastatin or resveratrol alone^29^.

Similarly, our study showed that administration of a vasodilatory drug, in this case uradipil, along with resveratrol resulted in alleviated cerebral damage in experimental cerebral ischemia reperfusion injury. Though urapidil was not evaluated in cerebral IR injury, it was shown to have anti-inflammatory, antioxidant, and antiapoptotic properties^11^^,^ 12. Güler et al.^11^ and Meštrović et al.^12^ independently showed that urapidil significantly decreased MDA level and significantly increased SOD activity in torsioned ovary and testis, respectively. Though urapidil in this study exhibited similar effects against IR, it was not pronounced as resveratrol. Nevertheless, addition of uradipil into treatment potentiated the effects of resveratrol.

Resveratrol was reported to reach its peak activity in brain tissue at 4 h in a previously published study on experimental cerebral stroke. It also downregulates glial fibrillary acidic protein (GFAP) immunopositivity and leads to an increased number of viable neurons^30^. A study by Yousuf et al.^31^ showed severe tissue injury in cerebral MCAO exposed animals such as swelling in pericellular cavity, and results of this study also indicated that the upregulated DNA fragmentation, lipid peroxidation, and oxidative stress are alleviated in resveratrol-treated animals. These pathological changes and beneficial effects of resveratrol were also observed in our study. Such improvements were also observed in urapidil-treated animals to some extent, but were less prominent than those that treated with resveratrol, indicating resveratrol is a more potent agent in this regard. On the other hand, higher degree of the most success for the recovery of brain morphology is obtained with combined treatment with resveratrol and urapidil.

Resveratrol, besides antioxidant effects, has antiapoptotic effects. Li et al.^31^ indicated that resveratrol treatment alleviated infarct volume, hippocampal apoptotic cell death ratio, and Bax expression in stroke exposed animals, and results of this study indicated that the suppressed Bcl-2 expression in MCAO group is improved in resveratrol-treated animals^32^. Urapidil has also shown to decrease apoptosis index in testicular torsion-detorsion injury in rats^12^. Additionally, Güler et al.^11^ found significant decrease in caspase-3 injury in urapidil-treated rats exposed to ovarian torsion detorsion injury. Our examinations indicated that resveratrol treatment was beneficial for not only anti-apoptotic Bcl-2, but also pro-apoptotic caspase-3 expression. So, we can conclude that resveratrol is successful in inhibiting the activated apoptotic signaling in stroke. Moreover, our analysis revealed the limited success of urapidil compared to resveratrol. Notably, the combined treatment emerged as the most beneficial, consistent with microscopic and histopathological examinations.

The studies indicate that resveratrol influences various signaling processes crucial for neuroprotection. For instance, it has been shown to modulate the apoptotic signaling pathway involving JAK2/STAT3/PI3K/AKT/mTOR, highlighting its potential in preventing cell death^33^. Additionally, resveratrol plays a role in the inflammatory JAK/ERK/STAT signaling pathway, suggesting its multifaceted impact on mitigating inflammation during neuroprotection^34^.

These findings underscore the complexity of the neuroprotective mechanisms associated with resveratrol administration. Fang et al.^35^’s report on downregulation of tissue TNF-α levels by resveratrol treatment in experimental stroke supported the anti-inflammatory effects of resveratrol. Similarly, we observed decreased levels of TNF-α in resveratrol-treated group. However, this effect on TNF-α was not as pronounced in urapidil-treated animals. Moreover, the combined treatment of resveratrol and urapidil notably suppressed TNF-α expression, further emphasizing its potential efficacy.

## Conclusion

Our study indicates that urapidil exhibits lower effectiveness in mitigating IR injury compared to resveratrol. Notably, the combined treatment of resveratrol and urapidil demonstrates a heightened degree of antioxidant, antiapoptotic, and anti-inflammatory effects when compared to the individual use of either drug. To validate these findings and establish optimal treatment dosages for the combined approach, further research is warranted.

## Data Availability

Data sharing is not applicable.

## References

[1] GBD 2019 Stroke Collaborators Global, regional, and national burden of stroke and its risk factors, 1990-2019 (2021). Global, regional, and national burden of stroke and its risk factors, 1990-2019: a systematic analysis for the Global Burden of Disease Study 2019. Lancet Neurol.

[2] Yuan Q, Yuan Y, Zheng Y, Sheng R, Liu L, Xie F, Tan J (2021). Anti-cerebral ischemia reperfusion injury of polysaccharides: A review of the mechanisms. Biomed Pharmacother.

[3] Guo J, Wang SB, Yuan TY, Wu YJ, Yan Y, Li L, Xu XN, Gong LL, Qin HL, Fang LH, Du GH (2013). Coptisine protects rat heart against myocardial ischemia/reperfusion injury by suppressing myocardial apoptosis and inflammation. Atherosclerosis.

[4] Seker U, Nergiz Y, Aktas A, Akkus M, Ozmen MF, Uyar E, Soker S (2020). Trolox is more successful than allopurinol to reduce degenerative effects of testicular ischemia/reperfusion injury in rats. J Pediatr Urol.

[5] Ishii T, Asai T, Oyama D, Agato Y, Fukuta T, Shimizu K, Minamino T, Oku N (2013). Treatment of cerebral ischemia-reperfusion injury with PEGylated liposomes encapsulating FK506. Faseb J.

[6] López-Morales MA, Castelló-Ruiz M, Burguete MC, Hervás D, Pérez-Pinzón MA, Salom JB (2023). Effect and mechanisms of resveratrol in animal models of ischemic stroke: A systematic review and Bayesian meta-analysis. J Cereb Blood Flow Metab.

[7] Berman AY, Motechin RA, Wiesenfeld MY, Holz MK (2017). The therapeutic potential of resveratrol: a review of clinical trials. NPJ Precis Oncol.

[8] Giovannini L, Migliori M, Longoni BM, Das DK, Bertelli A, Panichi V, Filippi C, Bertelli A (2001). Resveratrol, a polyphenol found in wine, reduces ischemia reperfusion injury in rat kidneys. J Cardiovasc Pharmacol.

[9] Najafi M, Tavakol S, Zarrabi A, Ashrafizadeh M (2022). Dual role of quercetin in enhancing the efficacy of cisplatin in chemotherapy and protection against its side effects: a review. Arch Physiol Biochem.

[10] Li Y, Chen F, Zhang J, Wang T, Wei X, Wu J, Feng Y, Dai Z, Wu Q (2013). Neuroprotective effect of resveratrol on ischemia/reperfusion injury in rats through TRPC6/CREB pathways. J Mol Neurosci.

[11] Güler MC, Tanyeli A, Erdoğan DG, Eraslan E, Çomaklı S, Polat E, Doğanay S (2021). Urapidil alleviates ovarian torsion detorsion injury via regulating oxidative stress, apoptosis, autophagia, and inflammation. Iran J Basic Med Sci.

[12] Meštrović J, Pogorelić Z, Drmić-Hofman I, Vilović K, Todorić D, Popović M (2017). Protective effect of urapidil on testicular torsion-detorsion injury in rats. Surg Today.

[13] Huang Y, Pan L, Wu T (2021). Improvement of cerebral ischemia-reperfusion injury by L-3-n-butylphthalide through promoting angiogenesis. Exp Brain Res.

[14] Placer ZA, Cushman LL, Johnson BC (1966). Estimation of product of lipid peroxidation (malonyl dialdehyde) in biochemical systems. Anal Biochem.

[15] Sagir S, Bayrak O, Turgut O, Allahverdi S, Ulusal H (2022). Peroxinitrite and Malondialdehyde as Biomarkers for Overactive Bladder. J Coll Physicians Surg Pak.

[16] Sedlak J, Lindsay RH (1968). Estimation of total, protein-bound, and nonprotein sulfhydryl groups in tissue with Ellman’s reagent. Anal Biochem.

[17] Misra HP, Fridovich I (1972). The role of superoxide anion in the autoxidation of epinephrine and a simple assay for superoxide dismutase. J Biol Chem.

[18] Kurt S, Koca RH, Hürkul MM, Seker U, Köroğlu A (2021). The antioxidant effect of Michauxia campanuloides on rat ovaries. J Hellenic Vet Med Soc.

[19] Tunik E, Ayaz E, Akpolat V, Nergiz Y, Isen K, Celik MS, Seker U (2013). Effects of pulsed and sinusoidal electromagnetic fields on MMP-2, MMP-9, collagen type IV and E-cadherin expression levels in the rat kidney: an immunohistochemical study. Anal Quant Cytopathol Histpathol.

[20] Ayaz H, Kaya S, Seker U, Nergiz Y (2023). Comparison of the anti-diabetic and nephroprotective activities of vitamin E, metformin, and Nigella sativa oil on kidney in experimental diabetic rats. Iran J Basic Med Sci.

[21] Seker U, Kaya S, Irtegun Kandemir, Sener D, Unay O, Nergiz O (2022). Effects of black cumin seed oil on oxidative stress and expression of membrane-cytoskeleton linker proteins, radixin, and moesin in streptozotocin-induced diabetic rat liver. Hepatol Forum.

[22] Çetin R, Bahadir S, Basar İ, Aslanoglu B, Kaya S, Güzel BC, Turan Y (2024). Supplementary tables. Zenodo.

[23] Siniscalchi A, Gallelli L, Malferrari G, Pirritano D, Serra R, Santangelo E, De Sarro G (2014). Cerebral stroke injury: the role of cytokines and brain inflammation. J Basic Clin Physiol Pharmacol.

[24] Gibson CL (2013). Cerebral ischemic stroke: is gender important?. J Cereb Blood Flow Metab.

[25] Zaheer Z, Robinson T, Mistri AK (2011). Thrombolysis in acute ischaemic stroke: an update. Ther Adv Chronic Dis.

[26] Akbas H, Ozden M, Kanko M, Maral H, Bulbul S, Yavuz S, Ozker E, Berki T (2005). Protective antioxidant effects of carvedilol in a rat model of ischaemia-reperfusion injury. J Int Med Res.

[27] Margaill I, Plotkine M, Lerouet D (2005). Antioxidant strategies in the treatment of stroke. Free Radic Biol Med.

[28] Frémont L (2000). Biological effects of resveratrol. Life Sci.

[29] Liu Y, Yang H, Jia G, Li L, Chen H, Bi J, Wang C (2018). The Synergistic Neuroprotective Effects of Combined Rosuvastatin and Resveratrol Pretreatment against Cerebral Ischemia/Reperfusion Injury. J Stroke Cerebrovasc Dis.

[30] Wang Q, Xu J, Rottinghaus GE, Simonyi A, Lubahn D, Sun GY, Sun AY (2002). Resveratrol protects against global cerebral ischemic injury in gerbils. Brain Res.

[31] Youseuf S, Atif F, Ahmad M, Hoda N, Ishrat T, Khan B, Islam F (2009). Resveratrol exerts its neuroprotective effect by modulating mitochondrial dysfunctions and associated cell death during cerebral ischemia. Brain Res.

[32] Li Z, Pang L, Fang F, Zhang G, Zhang J, Xie M, Wang L (2012). Resveratrol attenuates brain damage in a rat model of focal cerebral ischemia via up-regulation of hippocampal Bcl-2. Brain Res.

[33] Hou Y, Wang K, Wan W, Cheng Y, Pu X, Ye X (2018). Resveratrol provides neuroprotection by regulating the JAK2/STAT3/PI3K/AKT/mTOR pathway after stroke in rats. Genes Dis.

[34] Chang C, Zhao Y, Song G, She K. (2018). Resveratrol protects hippocampal neurons against cerebral ischemia-reperfusion injury via modulating JAK/ERK/STAT signaling pathway in rats. J Neuroimmunol.

[35] Fang L, Gao H, Zhang W, Zhang W, Wang Y (2015). Resveratrol alleviates nerve injury after cerebral ischemia and reperfusion in mice by inhibiting inflammation and apoptosis. Int J Clin Exp Med.

